# Empiric tuberculosis treatment in South African primary health care facilities - for whom, where, when and why: Implications for the development of tuberculosis diagnostic tests

**DOI:** 10.1371/journal.pone.0191608

**Published:** 2018-01-24

**Authors:** Kerrigan McCarthy, Katherine Fielding, Gavin J. Churchyard, Alison D. Grant

**Affiliations:** 1 The Aurum Institute; Johannesburg, South Africa; 2 School of Public Health, University of the Witwatersrand, Johannesburg, South Africa; 3 National Institute for Communicable Diseases, Johannesburg, South Africa; 4 TB Centre, London School of Hygiene & Tropical Medicine, London, United Kingdom; 5 Advancing Treatment and Care, South African Medical Research Council, Johannesburg, South Africa; 6 Africa Health Research Institute, School of Nursing and Public Health, University of KwaZulu-Natal, Durban, South Africa; Emory University, UNITED STATES

## Abstract

**Background:**

The extent and circumstances under which empiric tuberculosis (TB) treatment (treatment without microbiological confirmation at treatment initiation) is administered in primary health care settings in South Africa are not well described.

**Methods:**

We used data from a pragmatic evaluation of Xpert MTB/RIF in which persons undergoing TB investigations by PHC nurses were followed for six months. Following Xpert or smear-microscopy at enrolment, investigations for tuberculosis were undertaken at the discretion of health care workers. We identified persons whose TB treatment was initiated empirically (no microbiological confirmation at time of treatment initiation at a primary health care facility) and describe pathways to treatment initiation.

**Results:**

Of 4665 evaluable participants, 541 persons were initiated on treatment of whom 167 (31%) had negative sputum tests at enrolment. Amongst these 167, the median number of participant visits to health care providers prior to treatment initiation was 3 (interquartile range [IQR] 2–4). Chest radiography, sputum culture or hospital referral was done in 106/167 (63%). Reasons for TB treatment start were: 1) empiric (n = 82, 49%); 2) a positive laboratory test (n = 49, 29%); 3) referral and treatment start at a higher level of care (n = 28, 17%); and 4) indeterminable (n = 8, 5%). Empiric treatment accounted for 15% (82/541) of all TB treatment initiations and 1.7% (82/4665) of all persons undergoing TB investigations. Chest radiography findings compatible with TB (63/82 [77%]) were the basis for treatment initiation amongst the majority of empirically treated participants. Microbiological confirmation of TB was subsequently obtained for 11/82 (13%) empirically-treated participants. Median time to empiric treatment start was 3.9 weeks (IQR 1.4–11 weeks) after enrolment.

**Conclusion:**

Uncommon prescription of empiric TB treatment with reliance on chest radiography in a nurse-managed programme underscores the need for highly sensitive TB diagnostics suitable for point-of-care, and strong health systems to support TB diagnosis in this setting.

## Introduction

‘Empiric tuberculosis (TB) treatment’- defined as the administration of TB treatment to persons being evaluated for TB who do not have laboratory evidence of TB—is advised by WHO guidelines in resource-limited settings when ambulant HIV-positive persons with two negative sputum smear microscopy tests and chest radiography findings compatible with TB do not respond to broad-spectrum antimicrobial therapy[1]. While empiric TB treatment is not without risks (unnecessary administration of TB drugs with potential for adverse effects or delay in the diagnosis of conditions other than TB), it is appropriate in a context where the pre-test probability of TB is high, diagnostics have inadequate sensitivity and/or the consequences of withholding TB treatment are serious[2]. In South Africa, the National Department of Health (NDoH) provides algorithms for TB diagnosis which advise additional investigations when initial sputum smear microscopy or Xpert MTB/RIF (Xpert) tests for TB are negative and persons under investigation are co-infected with HIV[3]. These algorithms indicate that clinicians may commence TB treatment for HIV-positive persons when two sputum smear microscopy tests or a single sputum Xpert are negative for *Mycobacterium tuberculosis*, chest radiograph findings are compatible with TB, and symptoms do not respond to broad spectrum antibiotics.

Neither the extent, nor the circumstances under which empiric TB treatment is administered in primary health care (PHC) settings in South Africa are well described. We have previously demonstrated poor adherence to the algorithms for TB diagnosis in a cluster-randomised, pragmatic trial (XTEND) that evaluated Xpert *vs* smear microscopy for the diagnosis of TB[4]. Using data from XTEND, in which we followed over 4,000 persons undergoing TB investigations by PHC nurses for six months, we identified persons receiving empiric TB treatment and describe their pathways to treatment initiation, including results of investigations, health seeking efforts and time to treatment start.

## Study population and methods

### The XTEND trial–study design and TB investigation procedures

The XTEND (Xpert for TB—Evaluating a New Diagnostic) study was a pragmatic, two-arm, parallel, cluster-randomised trial evaluating the effect on mortality of Xpert implementation *vs* smear microscopy for the diagnosis of TB amongst persons being evaluated for TB in PHC facilities in South Africa[5]. Twenty laboratories servicing 40 PHCs across four provinces were randomised to receive Xpert as described[5]. XTEND study staff enrolled a systematic sample of clinic attendees being evaluated for TB. Personal identifiers, demographic and clinical data relevant to TB and mortality risk were collected at enrolment. XTEND study staff had no influence on clinical investigations nor management decisions made by PHC staff. At six months post enrolment, XTEND study staff abstracted data from patients’ clinic records, obtained participants’ sputum results and conducted patient interviews. XTEND study methods are described more completely elsewhere[5]. At participating clinics, nurses identified persons who required investigation for TB according to local screening practice. When initial sputum test results (Xpert or smear microscopy) were negative for TB, further investigations (sputum mycobacterial culture, chest radiography and therapeutic trial of antibiotics) or referral to hospital were made at the discretion of the PHC nurse according to routine local practice[3, 6]. In clinics using Xpert, the NDoH had recently trained nurses in the use of the Xpert algorithm for TB diagnosis[3], while in clinics using smear microscopy, no additional training was given as part of XTEND trial procedures.

### Inclusion criteria for the secondary data analysis and case definitions

Participants in the XTEND study with negative initial sputum results (smear microscopy or Xpert) at enrolment who were started on TB treatment during any stage of the 6-month follow-up were included in the analysis. Participants were defined as ‘index test negative’ if sputum tests conducted within four days of enrolment (two smear microscopy tests or one Xpert) were negative for TB. Participants who had a TB treatment prescription in their clinic notes or reported having started TB treatment at six-month patient interview were regarded has having started TB treatment. Positive HIV status was ascertained through self-report, or any of a documented HIV positive test, any CD4 count, HIV viral load test or ART prescription in the clinic record.

For these eligible XTEND participants, the course of events leading to TB treatment initiation was reconstructed from data sources described above; this substudy entailed additional record review at a later timepoint compared to that undertaken in the main XTEND analysis. Through evaluation of participants’ pathways, the reasons for TB treatment initiation of participants were inferred according to Table 1. Participants whose reason for TB treatment initiation was ‘clinical assessment’ or ‘chest radiography findings compatible with TB’ were defined as receiving empiric TB treatment.

**Table 1:** Criteria used to infer the reason for TB treatment start amongst persons enrolled in the XTEND trial who had a negative sputum test for TB at enrolment by Xpert or smear microscopy, but who were started on TB treatment during the six month period after enrolment.

Criteria used to infer the reason for TB treatment start	Inferred reason for TB treatment start	Was TB treatment given empirically?
A sputum specimen collected at PHC after enrolment was positive for TB by at least one of smear microscopy, Xpert or culture AND Sputum results were available to PHC clinicians at the time of TB treatment start (as defined by collection date of the sputum preceded the TB treatment start date by a minimum of 2 days (smear microscopy or Xpert) or 14 days [culture]) WITH OR WITHOUT Chest radiography and/or referral to higher level of care	Positive laboratory sputum test for TB	No
Extra-pulmonary TB diagnosed at a higher level of care (including pleural TB, TB of lymph nodes or miliary/disseminated TB) AND No documented positive laboratory test (smear microscopy, Xpert or culture) at the time of TB treatment start WITH OR WITHOUT Chest radiography	Extra-pulmonary TB	No for the purposes of this analysis, but uncertain as clinical notes not available for review
No documented positive laboratory test (smear microscopy, Xpert or culture) at the time of TB treatment start AND No evidence of chest radiography AND No referral to a higher level of care	Clinical assessment	Yes
Chest radiography documented within 7 days prior to TB treatment start AND No positive smear microscopy, Xpert or sputum culture results for *M*. *tuberculosis* were available before TB treatment initiation (within 2 days of the sputum collection date (smear microscopy or Xpert) or within 14 days (culture)	Chest radiography findings compatible with TB	Yes
Consultation at a hospital out-patient clinic or hospital admission at or immediately prior to TB treatment start AND No positive smear microscopy, Xpert or sputum culture results for *M*. *tuberculosis* were available before TB treatment initiation (within 2 days of the sputum collection date (smear microscopy or Xpert) or within 14 days (culture) AND No documentation that a chest radiograph had been taken or requested at PHC	Assessment at a higher level of care leading to TB treatment initiation	No for the purposes of this analysis, but uncertain as clinical notes not available for review
Insufficient data and/or patient pathway could not be reliably reconstructed	No reason assigned	Uncertain

### Institutional and ethical review

The XTEND protocol and this secondary data analysis received formal approval following review by the Human Sciences Research Ethics Committee (HREC) of the University of the Witwatersrand in 2011 (M110827), the London School of Hygiene & Tropical Medicine, the University of Cape Town and WHO. All XTEND participants provided written (name or thumbprint) informed consent for participation. In this secondary analysis which was approved by the University of the Witwatersrand in 2013 (M140486), all data were fully anonymized prior to analysis.

## Results

**Fig 1:**
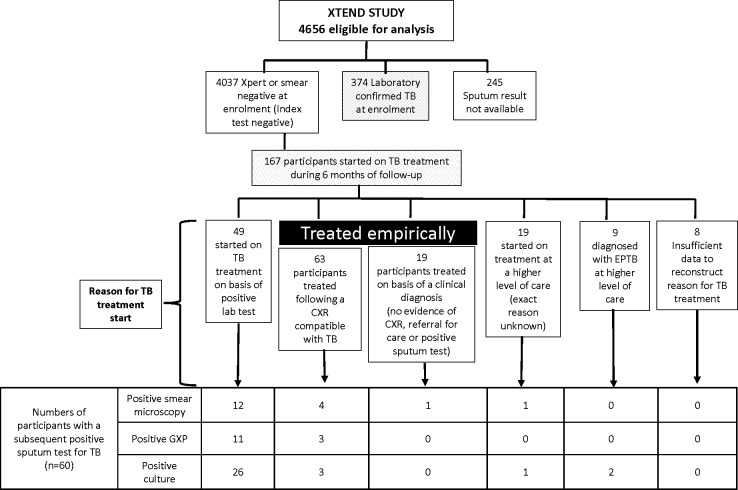
A study profile illustrating the reason for TB treatment start and results of subsequent sputum TB tests amongst persons investigated for TB whose index (enrolment) sputum was negative for TB when tested by Xpert or smear microscopy.

Amongst 4,656 XTEND participants, 541 (12%)[5] initiated TB treatment by six months of follow up of whom 167 (31%) were index test negative at enrolment, and were started on TB treatment during 6 months of follow-up. The median age was 37 years (31–45 years) and 79/167 (49%) were female. HIV status was positive at or before enrolment amongst 120/167 (72%). Regarding TB symptoms cough, fever, sweats or weight loss, the majority (109, 65%) had 2 or more weeks of symptoms prior to enrolment, and 3 or 4 symptoms (103, 62%) vs 1–2 or none. The median number of health seeking visits prior to enrolment was 1 (IQR = 0–2) with the majority having sought help from a public clinic (126, 75%). Table 2 describes the characteristics of: 1) all XTEND participants with negative sputum tests for tuberculosis at enrolment; 2) the subgroup who were started on TB treatment on an empiric basis; and 3) the subgroup with subsequent microbiological confirmation of tuberculosis. As some participants who were started on empiric TB treatment were subsequently found to have microbiologically confirmed TB, subgroups 2) and 3) are not mutually exclusive. **Fig** 1. depicts reasons for TB treatment start which were: 1) a positive sputum test for TB taken after enrolment (n = 49, 29%); 2) presumed chest radiography findings compatible with TB (n = 63, 38%); 3) clinical assessment suggestive of TB (n = 19, 11%); 4) following assessment at or admission to a higher level of care (n = 19, 11%), and 5) a diagnosis of extra-pulmonary TB (EPTB) made at a higher level of care (n = 9, 5%) or 6) reasons unclear (n = 8, 5%). A total of 82/167 (49%) were initiated on TB treatment empirically (I.e. did not have bacteriological confirmation when TB treatment was started), representing 15% (82/541) of treatment initiations in the entire XTEND cohort. Amongst 167 participants started on TB treatment, 60 (36%) had a subsequent positive sputum test for TB at PHC level (smear microscopy, Xpert or culture, before or after starting TB treatment (**Fig** 1). The remaining 107/541 (20%) XTEND participants were treated for TB without any microbiological confirmation of TB at PHC level (though the participants started at a higher level of care may have had laboratory tests confirming TB). Amongst 167 participants with negative TB tests at enrolment who were subsequently started on TB treatment, 113 (68%) were in the smear microscopy arm of the XTEND trial.

**Table 2:** Characteristics of XTEND participants with negative sputum tests for tuberculosis by smear microscopy or Xpert at enrolment into XTEND who were subsequently started on TB treatment during the 6-month follow-up (n = 167). Sub-groups 1 (empirically treated participants) and sub-group 2 (participants with microbiologically-confirmed TB) are not mutually exclusive as some participants who were started on empiric TB treatment were subsequently found to have microbiologically confirmed TB.

	All participants with negative sputum index results at enrolment who were subsequently started on TB treatment		
	Entire cohort N = 167	Sub-group 1: Empiric group* N = 82	Sub-group 2: Microbiologically confirmed TB^#^ N = 60
**Characteristics at enrolment**			
Female gender, n (%)	79 (47)	39 (48)	25 (41)
Age in years, median (IQR)	37 (31–45)	36 (30–45)	37 (31–43)
Residing in a rural area, n (%)	72 (43)	32 (39)	27 (45)
HIV status at enrolment			
HIV positive before or at enrolment/HIV status known, n/N, (%)	120/137 (88)	59/78 (76)	46/50 (92)
on ART, among those HIV positive, n/N (%)	30/120 (25)	10/59 (17)	14/46 (30)
Previous TB, n (%)	41 (25)	22/82 (27)	16 (26)
Karnofsky score <80, n (%)	41 (25)	46/82 (47)	16 (27)
Body mass index <18.5, kg/m^2^, n (%)	23 (14)	9 (11)	11 (18)
XTEND study arm			
Smear microscopy	113 (68)	61 (74)	43 (72)
Xpert	54 (32)	21 (26)	17 (28)
**Health seeking behaviour and symptoms prior to and at enrolment**			
Participants with duration of symptoms > 2 weeks prior to enrolment, n (%)	109 (65)	47 (57)	14 (23)
Enrolment visit was first health seeking visit for TB symptoms, n (%)	111 (66)	43 (52)	30 (50)
Visits to health care provider prior to enrolment, median (IQR)	1 (0–2)	1.5 (1–3)	1 (0–2)
Provider from whom participant first sought help for presenting symptoms			
Public clinic, n, %	126 (75)	66 (80)	46 (77)
Pharmacy, n (%)	20 (12)	8 (10)	9 (15)
Private doctor/other, n(%)	15 (9)	5 (7)	5 (8)
Public Hospital, n (%)	5 (3)	3 (5)	0
Traditional healer, n (%)	1 (1)	0	0
Reason for visit to PHC at which enrolment took place			
TB symptoms, n (%)	140 (84)	71 (87)	51 (85)
HIV testing or routine visit, n (%)	15 (9)	8 (8)	5(8)
Chronic disease routine visit/other, n (%)	12 (7)	3 (4)	4 (7)
Number of symptoms at enrolment			
None, n (%)	3 (2)	1 (1)	2 (3)
1–2, n (%)	61 (37)	29 (35)	22 (37)
3–4, n (%)	103 (62)	52 (64)	36 (60)

* A subset of the group n = 167 who met the definition for empiric TB treatment initiation as defined in Table 1. Eleven had a subsequent positive microbiology test on sputum.

# A subset of the group n = 167 who had a positive Xpert or smear microscopy or culture result before or after TB treatment start. Eleven were started on TB treatment empirically. IQR = interquartile range, PHC = primary health clinic

### Pathways to TB treatment, visits to health care providers and vital status at 6 months of follow-up

**Fig 2:**
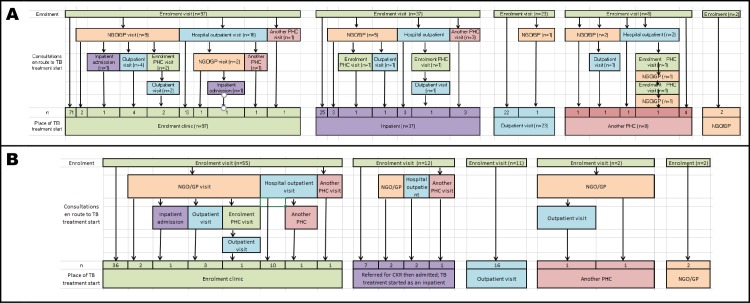
Consultations en route to TB treatment initiation amongst 167 XTEND participants with negative smear microscopy tests for TB at enrolment, reconstructed from data obtained from patient interviews, case note abstractions and laboratory results and stratified by TB treatment start. Sequential visits to the same provider are not shown. A: Pathways for entire cohort (n = 167). B: Pathways for persons started on TB treatment empirically (n = 82).

From enrolment to TB treatment start, the 167 participants completed 529 visits to health care providers to address TB symptoms including the enrolment visit, repeat visits to the same or different providers and the visit culminating in TB treatment start—a median of 3 visits per participant (interquartile range [IQR] 2–4). Table 3 summarises TB investigations, treatment initiation and vital status at 6 months follow up for all XTEND participants with negative sputum tests for tuberculosis and amongst the subgroups–those who were started on TB treatment on an empiric basis, and those who had subsequent microbiological confirmation of TB. Evidence for adherence to algorithms for TB investigation (any of documented chest radiography, sputum culture or referral to hospital) was found in 106 (63%). Documented or undocumented (self-reported) chest radiography was reported in 87/167 (52%). Sputum was submitted for culture amongst 64/167 (38%) participants of which 37/64 (58%) were positive for *M*. *tuberculosis*. TB treatment was started a median of 3.9 weeks (IQR 1.4–11 weeks) after enrolment amongst persons started empirically, and 8.6 weeks after enrolment amongst persons with microbiologically confirmed TB. Amongst 37 persons with culture-confirmed TB, 15 (41%) were initiated on TB treatment >8 weeks after sputum submission (median 15.6, range 8.4 to 31.7 weeks). **Fig 2** summarises the pathways to TB treatment during 6 months of follow-up grouped by place of TB treatment start and omitting multiple visits to the same provider. Amongst those who were started on TB treatment at their enrolment clinic and did not make use of other providers (n = 71), more than a third (26, 37%) made at least two subsequent visits to their enrolment clinic prior to TB treatment initiation. Amongst 60 participants who were initiated on TB treatment following assessment at a higher level of care, only 21 (33%) had a documented referral in the PHC notes. Over the 6 months of follow up, 12/167 (7%) participants died, of whom 11 were in the smear microscopy arm. Death occurred a median of 9 weeks (range 3–34 weeks) after enrolment and a median 3 weeks (range 0.8–10 weeks) after TB treatment initiation.

**Table 3:** TB investigations, treatment initiation and vital status at 6 months follow up of XTEND participants with negative sputum tests for tuberculosis by smear microscopy or Xpert who were subsequently started on TB treatment during the 6-month follow-up (n = 167). Sub-groups 1 (empirically treated participants) and sub-group 2 (participants with microbiologically-confirmed TB) are not mutually exclusive as some participants who were started on empiric TB treatment were subsequently found to have microbiologically confirmed TB.

	All participants with negative sputum index results at enrolment who were subsequently started on TB treatment		
	Entire cohort N = 167	Sub-group 1 Empiric group*^1^ N = 82	Sub-group 2 Microbiologically confirmed TB ^#^ N = 60
**Investigations for TB initiated by PHC staff after enrolment in XTEND**			
Sputum culture done, n (%)	64 (38)	23 (28)	42 (70)
Documented chest radiography, n (%)	58 (35)	51 (62)	9 (15)
Undocumented chest radiography, n (%) (patient reported at interview)	29 (17)	10 (11)	6 (10)
Any admission to hospital, n (%)	27 (22)	70 (85)	9 (15)
Algorithm followed (any of documented CXR, culture, or referral for hospital), n (%)	106 (63)	58 (71)	45 (75)
**TB treatment initiation**			
Reason for TB treatment start (as defined in Table 1)			
Clinical assessment compatible with TB, n (%)	19 (11)	19 (23)	1 (2)
Chest radiography compatible with TB (documented or undocumented), n (%)	63 (38)	63 (77)	10 (17)
Positive sputum test for TB^z^, n (%)	49 (29)	0	45 (75)
Extra-pulmonary TB diagnosed at higher level of care, n (%)	9 (5)	0	2 (3)
Assessment at a higher level of care leading to TB treatment initiation, n (%)	19 (11)	0	2 (3)
Unclear^y^, n (%)	8 (5)	0	0
TB treatment started ‘empirically’ (i.e. without laboratory confirmation at the time of TB treatment start), n (%)	82 (49)	82 (100)	11 (19)
Time from enrolment to TB treatment start in weeks, median (IQR)	5.7(2.1–12.1)	3.9 (1.4–11)	8.57 (3.9–15.3)
Place of TB treatment start			
Original clinic, n (%)	97 (58)	55 (67)	38 (63)
Another PHC clinic, n (%)	8 (5)	2 (2)	5 (8)
Private GP, n (%)	2 (1)	2 (2)	0
Hospital (OPD or ward), n (%)	60 (36)	23 (29)	17 (28)
**Laboratory results, ART care and outcome**			
Microbiologically-confirmed TB			
Any microbiological confirmation, n (%)	60 (36)	11 (13)	60 (100)
Positive smear microscopy, n (%)	12 (7)	5 (6)	12 (20)
Positive Xpert, n (%)	11 (6)	3 (4)	11 (18)
Positive culture, n (%)	37 (22)	3 (4)	37 (62)
**HIV testing and ART initiation**			
Final HIV and ART status (after 6 months of follow up)			
HIV positive, n (%)	129 (77)	65 (79)	49 (82)
HIV positive and on ART, n (%)	109 (65)	56 (68)	43 (72)
HIV negative (documented negative result), n (%)	30 (18)	15 (18)	10 (17)
Unknown, n (%)	8 (5)	2 (2)	1 (2)
Timing of TB treatment in relation to ART amongst HIV positive participants who were on ART at enrolment or initiated ART during follow-up			
TB Rx start before ART, n/N (%)	46/109 (42)	29/56 (52)	18/43 (42)
TB start within 3 months after ART, n/N (%)	19/109 (17)	8/56 (14)	8/43 (19)
TB start >3months after ART, n/N (%)	44/109 (40)	20/56 (36)	17/43 (39)
Death during follow-up, n (%)	12 (7)	5 (6)	3 (5)

*A subset of the group n = 167 who were started on TB treatment who met the definition for empiric TB treatment initiation as defined in Table 1

^1^empiric = without a positive microbiology test at the time of TB treatment start;

#A subset of the group n = 167 who had negative sputum result(s) at enrolment but after further investigation had a positive Xpert or smear microscopy or culture result. Some cases in this group (n = 11) are also amongst those that were started on TB treatment empirically as defined in Table 1;

PHC = primary health clinic, GP = general practitioner/private doctor, OPD = out patient department), CXR = chest X ray, IQR = interquartile range, ART = antiretroviral therapy;

### HIV care among participants with negative TB tests at enrolment who were subsequently started on TB treatment

Amongst 167 participants, 120 were documented to be HIV positive and 30 on ART at or before enrolment and an additional 9 tested HIV positive during the 6 months of follow up. Amongst all HIV positive participants, 79 (61%) initiated ART during follow-up, with 109 on ART by 6 months post enrolment. Amongst 129 HIV-positive persons initiated on TB treatment, microbiologically-confirmed TB was diagnosed in 49 (43%), of whom 18 (37%) had started TB treatment before ART initiation. Eight (15%) HIV-positive participants with microbiologically-confirmed-TB started treatment within 3 months after ART initiation and 18 (35%) started TB treatment three or more months after ART initiation.

## Discussion

In a prospective observational cohort of over 4,000 persons, of whom over 60% were HIV positive, investigated for TB at the discretion of clinic staff in South African PHC facilities[5], and amongst whom 541 (12%) initiated TB treatment, we report infrequent use of empiric TB treatment (15%, 82/541). Chest radiography was the leading investigation prompting empiric TB treatment initiation. This, and our observation of repeated health-seeking attempts at different providers and delayed TB treatment start when TB diagnosis amongst persons initially Xpert- or smear-negative was made by sputum culture highlight 1) the importance of chest radiography as a follow-on diagnostic test for tuberculosis even in the Xpert era, and 2) the need to strengthen PHC TB diagnostic processes including improved diagnostic tests, adherence to diagnostic algorithms, and follow-up of patients who remain symptomatic, or at high risk of death (those with unknown HIV status or HIV+ and not on ART)[5].

Our previous finding of poor adherence to TB diagnostic algorithms[4] and in this analysis, low frequency of empiric TB treatment initiation in a predominantly nurse-managed, PHC TB treatment programme demonstrate an on-going reliance on sputum TB diagnostic tests. While empiric TB treatment, delivered as per WHO guidelines for the management of smear-negative TB[1], is a rational and evidence-based intervention that reduces mortality amongst HIV-positive persons with negative initial sputum tests for tuberculosis[7–9], in a predominantly nurse-managed TB control programme we and others[10–12] have found that it may not be widely prescribed. Adherence to algorithms for TB diagnosis amongst persons with negative initial sputum TB tests is not always optimal[11, 13] nor possible. Chest radiography remains inaccessible for many, even in South Africa, and requires interpretation by doctors and functioning health referral networks. Access to sputum culture is often limited. Multiple visits to health facilities are required leading to increased patient costs[14, 15], and follow up of patients may be limited. Initiation of TB treatment on the basis of clinical assessment alone, while appropriate in certain contexts[16], is infrequently prescribed. These observations, together with autopsy data showing that the most frequent cause of death amongst HIV-positive persons is undiagnosed tuberculosis[17–19], suggest that empiric TB treatment could appropriately be more frequently prescribed amongst HIV-positive persons.

In our evaluation, the reliance on chest radiography as the reason for TB treatment initiation amongst persons being evaluated for TB with negative Xpert sputum tests is in keeping with findings from studies evaluating routine TB screening[20, 21] and diagnostic algorithms using Xpert[22–25]. In the TB-NEAT evaluation of Xpert, where all participants received smear microscopy or Xpert, and chest radiography and sputum culture, TB treatment was initiated empirically on the basis of chest radiographic findings in 313 (51%) of all 611 persons initiated on TB treatment[26]. In a multi-centre, individually randomised trial of Xpert vs smear-microscopy with pragmatic implementation of further diagnostic tests, liberal use of chest radiography was a major factor driving empiric TB treatment initiation[13].

We observed that empiric TB treatment was frequently initiated following a visit to hospital or during admission, but only a third of persons had a documented referral suggesting that the majority of patients self-referred to a higher level of care. This suggests that patients may lack faith in the ability of clinics to assist them, and that PHC facilities miss opportunities to conduct further investigations. Naidoo et al[27] reported poor perceptions of public sector services amongst South African TB patients. Further, weak case management such as failure to adhere to testing algorithms[27, 28] and sputum results not being available[27] may lead to missed opportunities to conduct additional investigations and delay TB treatment start[15]. Our observation of delayed treatment start amongst persons diagnosed by culture suggests failure to promptly recall patients with positive results, a finding reported by others[27]. Ultimately, health system performance including district and facility level support, continuity and quality of care provide the context for TB case management and impact directly on TB programme outcomes[29]. Churchyard[5], Creswell[30] and others[31, 32] attribute the failure of Xpert to improve patient outcomes, despite the improved sensitivity of Xpert to health system weaknesses in the TB and HIV continuum of care, with missed opportunities to diagnose and link persons into TB treatment.

Our findings underscore the need for highly sensitive TB diagnostic tests. Currently available diagnostic assays include the Xpert MTB/RIF Ultra cartridge, the loop-mediated amplification (LAMP) assay and urine lipoarabinomannan (LAM) tests. While Xpert MTB/RIF Ultra has demonstrated improved sensitivity[33], and a potential impact on mortality in high TB/HIV prevalent regions[34], the assay has a marginal reduction in specificity especially amongst persons with a prior TB treatment history[35]. The WHO advises that the LAMP assay may be used as a replacement for or follow-on test after smear-microscopy, but should not replace Xpert MTB/RIF where this is available[36]. The currently-available urine LAM assay has limited clinical applicability, and is recommended for use amongst persons with HIV infection and low CD4 counts (<100 cells/μl)[37].

The strength of our evaluation arises through the pragmatic XTEND study design with implementation of TB diagnosis in a real-world setting in 40 PHCs across four South African provinces, allowing follow up of persons investigated for TB for 6 months after submitting sputum for TB investigation. Our findings may be limited by possible poor clinical record keeping at clinics. We were not able to conduct patient record review at referral hospitals. In addition, the XTEND study design did not allow for confirmation of TB diagnoses amongst all participants, and so it was not possible to confirm TB diagnoses amongst persons who did not have sputum submitted for TB culture, or who died during follow-up.

## Conclusions

In this secondary data analysis of a cohort of symptomatic persons investigated for TB at primary health care level and followed up for 6 months, we observed uncommon use of empiric TB treatment, reliance on chest radiography and self-referral to hospital to initiate TB treatment when initial sputum tests were negative. In the context of high rates of death due to autopsy-confirmed previously undiagnosed TB in the South African context, our findings underscore the importance of highly sensitive TB diagnostics and strong health systems to support TB diagnosis in this setting.

## Supporting information

S1 Appendix Pathways from enrolment to end of follow up of 167 persons who were index sputum test negative for TB at enrolment, but who were started on TB treatment during 6 months of follow up. (XLSX)

## References

[1] WHO. Improving the diagnosis and treatment of smear-negative pulmonary and extra-pulmonary tuberculosis among adults and adolescents: Recommendations for HIV-prevalent and resource-constrained settings. Geneva, Switzerland2007.

[2] Theron G, Peter J, Dowdy D, Langley I, Squire SB, Dheda K. Do high rates of empirical treatment undermine the potential effect of new diagnostic tests for tuberculosis in high-burden settings? Lancet Infect Dis. 2014;14(6):527–32. Epub 2014/01/21. doi: 10.1016/S1473-3099(13)70360-8 . 24438820 10.1016/S1473-3099(13)70360-8

[3] National Department of Health. National Tuberculosis Management Guidelines Tshwane2014.

[4] McCarthy KM, Grant AD, Chihota V, Ginindza S, Mvusi L, Churchyard GJ, et al Implementation and Operational Research: What Happens After a Negative Test for Tuberculosis? Evaluating Adherence to TB Diagnostic Algorithms in South African Primary Health Clinics. Journal of acquired immune deficiency syndromes (1999). 2016;71(5):e119–26. Epub 2016/03/12. doi: 10.1097/qai.0000000000000907 ; PubMed Central PMCID: PMCPmc4804742. 26966843 10.1097/QAI.0000000000000907 PMC4804742

[5] Churchyard GJ, Stevens WS, Mametja LD, McCarthy KM, Chihota V, Nicol MP, et al Xpert MTB/RIF versus sputum microscopy as the initial diagnostic test for tuberculosis: a cluster-randomised trial embedded in South African roll-out of Xpert MTB/RIF. Lancet Glob Health. 2015;3(8):e450–7. Epub 2015/07/19. doi: 10.1016/S2214-109X(15)00100-X . 26187490 10.1016/S2214-109X(15)00100-X

[6] National Department of Health. National Tuberculosis Management Guidelines. Tshwane 2009.

[7] Holtz TH, Kabera G, Mthiyane T, Zingoni T, Nadesan S, Ross D, et al Use of a WHO-recommended algorithm to reduce mortality in seriously ill patients with HIV infection and smear-negative pulmonary tuberculosis in South Africa: an observational cohort study. Lancet Infect Dis. 2011;11(7):533–40. Epub 2011/04/26. doi: 10.1016/S1473-3099(11)70057-3 . 21514234 10.1016/S1473-3099(11)70057-3

[8] Nakiyingi L, Nonyane BA, Ssengooba W, Kirenga BJ, Nakanjako D, Lubega G, et al Predictors for MTB Culture-Positivity among HIV-Infected Smear-Negative Presumptive Tuberculosis Patients in Uganda: Application of New Tuberculosis Diagnostic Technology. PloS One. 2015;10(7):e0133756 Epub 2015/07/30. doi: 10.1371/journal.pone.0133756 ; PubMed Central PMCID: PMCPmc4519276. 26222142 10.1371/journal.pone.0133756 PMC4519276

[9] Walusimbi S, Bwanga F, De Costa A, Haile M, Joloba M, Hoffner S. Meta-analysis to compare the accuracy of GeneXpert, MODS and the WHO 2007 algorithm for diagnosis of smear-negative pulmonary tuberculosis. BMC Infect Dis. 2013;13:507 Epub 2013/11/01. doi: 10.1186/1471-2334-13-507 ; PubMed Central PMCID: PMCPmc3833313. 24172543 10.1186/1471-2334-13-507 PMC3833313

[10] Loveday M, Thomson L, Chopra M, Ndlela Z. A health systems assessment of the KwaZulu-Natal tuberculosis programme in the context of increasing drug resistance. Int J Tuberc Lung Dis. 2008;12(9):1042–7. Epub 2008/08/21. . 18713502

[11] Tafuma TA, Burnett RJ, Huis In 't Veld D. National guidelines not always followed when diagnosing smear-negative pulmonary tuberculosis in patients with HIV in Botswana. PloS One. 2014;9(2):e88654 Epub 2014/02/20. doi: 10.1371/journal.pone.0088654 ; PubMed Central PMCID: PMCPmc3925109. 24551128 10.1371/journal.pone.0088654 PMC3925109

[12] Alamo ST, Kunutsor S, Walley J, Thoulass J, Evans M, Muchuro S, et al Performance of the new WHO diagnostic algorithm for smear-negative pulmonary tuberculosis in HIV prevalent settings: a multisite study in Uganda. Trop Med Int Health. 2012;17(7):884–95. Epub 2012/05/12. doi: 10.1111/j.1365-3156.2012.03003.x . 22575012 10.1111/j.1365-3156.2012.03003.x

[13] Calligaro GL, Zijenah LS, Peter JG, Theron G, Buser V, McNerney R, et al Effect of new tuberculosis diagnostic technologies on community-based intensified case finding: a multicentre randomised controlled trial. Lancet Infect Dis. 2017;17(4):441–50. Epub 2017/01/09. doi: 10.1016/S1473-3099(16)30384-X . 28063795 10.1016/S1473-3099(16)30384-X

[14] Foster N, Vassall A, Cleary S, Cunnama L, Churchyard G, Sinanovic E. The economic burden of TB diagnosis and treatment in South Africa. Soc Sci Med. 2015;130:42–50. Epub 2015/02/15. doi: 10.1016/j.socscimed.2015.01.046 . 25681713 10.1016/j.socscimed.2015.01.046

[15] Shete PB, Haguma P, Miller CR, Ochom E, Ayakaka I, Davis JL, et al Pathways and costs of care for patients with tuberculosis symptoms in rural Uganda. Int J Tuberc Lung Dis. 2015;19(8):912–7. Epub 2015/07/15. doi: 10.5588/ijtld.14.0166 . 26162356 10.5588/ijtld.14.0166 PMC6602531

[16] Griesel R, Stewart A, van der Plas H. A clinical prediction rule for the diagnosis of tuberculosis in seriously ill adults. Abstract #742, Conference on Retroviruses and Opportunistic Infections; Boston, February 22–252016.

[17] Karat AS, Omar T, von Gottberg A, Tlali M, Chihota VN, Churchyard GJ, et al Autopsy Prevalence of Tuberculosis and Other Potentially Treatable Infections among Adults with Advanced HIV Enrolled in Out-Patient Care in South Africa. PloS One. 2016;11(11):e0166158 Epub 2016/11/10. doi: 10.1371/journal.pone.0166158 ; PubMed Central PMCID: PMCPMC5102350. 27829072 10.1371/journal.pone.0166158 PMC5102350

[18] Wong EB, Omar T, Setlhako GJ, Osih R, Feldman C, Murdoch DM, et al Causes of death on antiretroviral therapy: a post-mortem study from South Africa. PloS One. 2012;7(10):e47542 Epub 2012/10/25. doi: 10.1371/journal.pone.0047542 ; PubMed Central PMCID: PMCPMC3472995. 23094059 10.1371/journal.pone.0047542 PMC3472995

[19] Maraba N, Karat AS, McCarthy K, Churchyard GJ, Charalambous S, Kahn K, et al Verbal autopsy-assigned causes of death among adults being investigated for TB in South Africa. Trans R Soc Trop Med Hyg. 2016;110(9):510–6. Epub 2016/10/30. doi: 10.1093/trstmh/trw058 ; PubMed Central PMCID: PMCPMC5091329. 27794093 10.1093/trstmh/trw058 PMC5091329

[20] Getahun H, Kittikraisak W, Heilig CM, Corbett EL, Ayles H, Cain KP, et al Development of a standardized screening rule for tuberculosis in people living with HIV in resource-constrained settings: individual participant data meta-analysis of observational studies. PLoS Med. 2011;8(1):e1000391 Epub 2011/01/27. doi: 10.1371/journal.pmed.1000391 ; PubMed Central PMCID: PMCPMC3022524. 21267059 10.1371/journal.pmed.1000391 PMC3022524

[21] Modi S, Cavanaugh JS, Shiraishi RW, Alexander HL, McCarthy KD, Burmen B, et al Performance of Clinical Screening Algorithms for Tuberculosis Intensified Case Finding among People Living with HIV in Western Kenya. PloS One. 2016;11(12):e0167685 Epub 2016/12/10. doi: 10.1371/journal.pone.0167685 ; PubMed Central PMCID: PMCPMC5147932. 27936146 10.1371/journal.pone.0167685 PMC5147932

[22] Creswell J, Codlin AJ, Andre E, Micek MA, Bedru A, Carter EJ, et al Results from early programmatic implementation of Xpert MTB/RIF testing in nine countries. BMC Infect Dis. 2014;14:2 Epub 2014/01/05. doi: 10.1186/1471-2334-14-2 ; PubMed Central PMCID: PMCPmc3898850. 24383553 10.1186/1471-2334-14-2 PMC3898850

[23] Muyoyeta M, Maduskar P, Moyo M, Kasese N, Milimo D, Spooner R, et al The sensitivity and specificity of using a computer aided diagnosis program for automatically scoring chest X-rays of presumptive TB patients compared with Xpert MTB/RIF in Lusaka Zambia. PloS One. 2014;9(4):e93757 Epub 2014/04/08. doi: 10.1371/journal.pone.0093757 ; PubMed Central PMCID: PMCPmc3976315. 24705629 10.1371/journal.pone.0093757 PMC3976315

[24] Muyoyeta M, Moyo M, Kasese N, Ndhlovu M, Milimo D, Mwanza W, et al Implementation Research to Inform the Use of Xpert MTB/RIF in Primary Health Care Facilities in High TB and HIV Settings in Resource Constrained Settings. PloS One. 2015;10(6):e0126376 Epub 2015/06/02. doi: 10.1371/journal.pone.0126376 ; PubMed Central PMCID: PMCPmc4451006. 26030301 10.1371/journal.pone.0126376 PMC4451006

[25] Theron G, Pooran A, Peter J, van Zyl-Smit R, Kumar Mishra H, Meldau R, et al Do adjunct tuberculosis tests, when combined with Xpert MTB/RIF, improve accuracy and the cost of diagnosis in a resource-poor setting? Eur Respir J. 2012;40(1):161–8. Epub 2011/11/15. doi: 10.1183/09031936.00145511 . 22075479 10.1183/09031936.00145511 PMC5523948

[26] Theron G, Zijenah L, Chanda D, Clowes P, Rachow A, Lesosky M, et al Feasibility, accuracy, and clinical effect of point-of-care Xpert MTB/RIF testing for tuberculosis in primary-care settings in Africa: a multicentre, randomised, controlled trial. Lancet. 2014;383(9915):424–35. Epub 2013/11/02. doi: 10.1016/S0140-6736(13)62073-5 . 24176144 10.1016/S0140-6736(13)62073-5

[27] Naidoo P, van Niekerk M, du Toit E, Beyers N, Leon N. Pathways to multidrug-resistant tuberculosis diagnosis and treatment initiation: a qualitative comparison of patients' experiences in the era of rapid molecular diagnostic tests. BMC Health Serv Res. 2015;15:488 Epub 2015/10/30. doi: 10.1186/s12913-015-1145-0 ; PubMed Central PMCID: PMCPmc4624595. 26511931 10.1186/s12913-015-1145-0 PMC4624595

[28] Cattamanchi A, Miller CR, Tapley A, Haguma P, Ochom E, Ackerman S, et al Health worker perspectives on barriers to delivery of routine tuberculosis diagnostic evaluation services in Uganda: a qualitative study to guide clinic-based interventions. BMC Health Serv Res. 2015;15:10 Epub 2015/01/23. doi: 10.1186/s12913-014-0668-0 ; PubMed Central PMCID: PMCPmc4307676. 25609495 10.1186/s12913-014-0668-0 PMC4307676

[29] Loveday M, Padayatchi N, Wallengren K, Roberts J, Brust JC, Ngozo J, et al Association between health systems performance and treatment outcomes in patients co-infected with MDR-TB and HIV in KwaZulu-Natal, South Africa: implications for TB programmes. PloS One. 2014;9(4):e94016 Epub 2014/04/11. doi: 10.1371/journal.pone.0094016 ; PubMed Central PMCID: PMCPmc3981751. 24718306 10.1371/journal.pone.0094016 PMC3981751

[30] Creswell J, Rai B, Wali R, Sudrungrot S, Adhikari LM, Pant R, et al Introducing new tuberculosis diagnostics: the impact of Xpert((R)) MTB/RIF testing on case notifications in Nepal. Int J Tuberc Lung Dis. 2015;19(5):545–51. Epub 2015/04/14. doi: 10.5588/ijtld.14.0775 . 25868022 10.5588/ijtld.14.0775

[31] Mbonze NB, Tabala M, Wenzi LK, Bakoko B, Brouwer M, Creswell J, et al Xpert((R)) MTB/RIF for smear-negative presumptive TB: impact on case notification in DR Congo. Int J Tuberc Lung Dis. 2016;20(2):240–6. Epub 2016/01/23. doi: 10.5588/ijtld.15.0177 . 26792478 10.5588/ijtld.15.0177

[32] Hanrahan CF, Haguma P, Ochom E, Kinera I, Cobelens F, Cattamanchi A, et al Implementation of Xpert MTB/RIF in Uganda: Missed Opportunities to Improve Diagnosis of Tuberculosis. Open Forum Infect Dis. 2016;3(2):ofw068 Epub 2016/05/18. doi: 10.1093/ofid/ofw068 ; PubMed Central PMCID: PMCPmc4866550. 27186589 10.1093/ofid/ofw068 PMC4866550

[33] Dorman SE, Schumacher SG, Alland D, Nabeta P, Armstrong DT, King B, et al Xpert MTB/RIF Ultra for detection of Mycobacterium tuberculosis and rifampicin resistance: a prospective multicentre diagnostic accuracy study. Lancet Infect Dis. 2017 Epub 2017/12/05. doi: 10.1016/s1473-3099(17)30691-6 . 29198911 10.1016/S1473-3099(17)30691-6 PMC6168783

[34] Kendall EA, Schumacher SG, Denkinger CM, Dowdy DW. Estimated clinical impact of the Xpert MTB/RIF Ultra cartridge for diagnosis of pulmonary tuberculosis: A modeling study. PLoS Med. 2017;14(12):e1002472 Epub 2017/12/15. doi: 10.1371/journal.pmed.1002472 ; PubMed Central PMCID: PMCPMC5730108. 29240766 10.1371/journal.pmed.1002472 PMC5730108

[35] WHO. WHO meeting report of a technical expert consultation: Non-inferiority analysis of Xpert MTB/RIF Ultra compared to Xpert MTB/RIF. Geneva, Switzerland: 2017.

[36] WHO. The use of loop mediated isothermal amplification (TB-LAMP) for the diagnosis of pulmonary tuberculosis: policy guidance. Geneva, Switzerland: WHO, 2016. 27606385

[37] WHO. The use of lateral flow urine lipoarabinomannan assay (LF-LAM) for the diagnosis and screening of active tuberculosis in peole living with HIV. Policy update. Geneva, Switzerland: WHO, 2015.

